# The Renin-Angiotensin System in the Development of Salt-Sensitive Hypertension in Animal Models and Humans

**DOI:** 10.3390/ph3040940

**Published:** 2010-03-29

**Authors:** Beate Rassler

**Affiliations:** Carl-Ludwig-Institute of Physiology, University of Leipzig, Liebigstr. 27, 04103 Leipzig, Germany; E-Mail: beate.rassler@medizin.uni-leipzig.dep; Tel.: +49-341-971-5514; Fax: +49-341-971-5509

**Keywords:** hypertension, salt sensitivity, renin-angiotensin system, antihypertensive treatment

## Abstract

Hypertension is still one of the major causes of death from cardiovascular failure. Increased salt intake may aggravate the rise in blood pressure and the development of consequential damage of the heart, the vessels and other organs. The general necessity of restricted salt intake regardless of blood pressure or salt sensitivity has been a matter of debate over the past decades. This review summarizes the main pathogenic mechanisms of hypertension and salt sensitivity in rat models, particularly in the spontaneously hypertensive rat (SHR), and in patients with essential hypertension (EH). Although SHRs are commonly considered to be salt-resistant, there is much evidence that salt loading may deteriorate blood pressure and cardiovascular function even in these animals. Similarly, EH is not a homogenous disorder – some patients, but not all, exhibit pronounced salt sensitivity. The renin-angiotensin system (RAS) plays a key role in the regulation of blood pressure and salt and fluid homeostasis and thus is one of the main targets of antihypertensive therapy. This review focuses on the contribution of the RAS to the pathogenesis of salt-sensitive hypertension in SHRs and patients with EH.

## 1. Introduction

Essential hypertension (EH) comprises about 90-95% of all cases of hypertension in humans. The genetic contribution to blood pressure (BP) variations in humans is estimated to be in the range of 30–50% [1]. In the majority of these patients, hypertension has a multigenetic base and exhibits heterogenous features. In particular, expression of salt sensitivity differs widely, subdividing EH patients into salt-sensitive and salt-resistant types. In the literature, statements concerning the percentage of salt-sensitive EH patients are inconsistent ranging between 30% and 70% [2,3].

Animal models of genetic hypertension are often used to study the mechanisms of hypertension and salt sensitivity in EH. The most common animal model in this respect is the spontaneously hypertensive rat (SHR). A large part of the findings discussed in the present review has been obtained in this model. Several mechanisms, however, have only been studied in other rat strains such as salt-sensitive Dahl (Dahl S) or Milan hypertensive rats but not in SHR and vice versa. SHRs are not considered to be specifically salt-sensitive, although it has been demonstrated repeatedly that high salt intake can increase their BP [4,5]. Rats also respond to elevated salt intake with enhanced consequential organ damage such as cardiac and vascular hypertrophy, remodelling and loss of function [6,7,8,9]. However, the increase in BP following high salt exposure is not limited to humans and animals with manifest hypertension. Young SHRs at a pre-hypertensive stage of life presented a greatly enhanced pressor response to salt loading by intracerebroventricular (ICV) injection of hypertonic saline [10]. Even normotensive organisms may exhibit salt sensitivity of BP. Monitoring of diurnal variations in mean arterial pressure (MAP) under high salt exposure revealed elevated MAP in normotensive controls (Wistar-Kyoto rats, WKY) during the night but not during the day, so that their circadian average was normal [4]. SHRs responded to this high-salt diet with continuous elevation in MAP. This differential reaction indicates that WKY are able to compensate for salt loading, thus preventing a sustained increase in mean arterial pressure, while compensation is deficient in genetic hypertensive animals [4].

Salt sensitivity in hypertensive patients is an important problem in a society with relatively high salt consumption as is the case in many developed countries. Hypertensive patients are recommended to follow a strict low-salt diet as a supplement to antihypertensive therapy. However, many patients are not willing to lower their salt intake, although increased salt consumption may compromise the therapeutic effects of antihypertensive drugs. In a study on SHRs at increased salt intake, several pharmacological lines of antihypertensive treatment failed to reduce blood pressure, despite the fact that the chosen drug doses had been reported to be sufficient for hypotensive effects at normal salt intake [11]. 

There is abundant evidence that salt sensitivity has a genetic base. Numerous quantitative trait loci (QTL) have been found to be associated with increased salt sensitivity in patients with EH (for a systematic review see [12]) as well as in SHRs [13,14,15,16]. Among the manifold candidate genes there is a large number of genes coding for components of the renin-angiotensin system (RAS) such as angiotensinogen [17], angiotensin-converting enzyme (ACE) [18,19], renin [20], or aldosterone synthase [21]. Additionally, various other genes associated with salt-sensitive hypertension affect products that are indirectly related to RAS function, e.g. adducin or dopamine [22,23,24]. 

Studies in rats have shown that the pathogenesis of genetic forms of hypertension, and especially of salt sensitivity, is largely based on elevated activity of the RAS and of the sympathetic nervous system (SNS) [25,26,27]. Many of the processes initiating a sustained increase in BP are localized in the brain. Elevated BP results predominantly from impaired sodium regulation and increased vasomotor tone. Besides the brain, the kidney and the cardiovascular system play a crucial role in the pathogenesis of hypertension. 

This review summarizes the main pathogenic processes involved in the development of salt-sensitive hypertension in humans and animal models with genetic hypertension. In particular, the role of the SNS, of circulating and local RAS and of modulators of the sodium-potassium ATPase (Na/K-ATPase) activity is considered. The review focuses on the brain, the kidney and the cardiovascular system in terms of the effects of high salt exposure on blood pressure and on consequential damage to the cardiovascular system. It emphasizes the pivotal function of the RAS that justifies the importance of RAS antagonists such as blockers of the angiotensin receptor type 1 (AT1) or inhibitors of the angiotensin converting enzyme (ACEI) in the treatment of essential hypertension. 

## 2. Pathogenetic Processes in the Development of Salt Sensitivity and Hypertension

### 2.1. Brain

The central nervous system plays a pivotal role in the development of salt-sensitive hypertension. Abnormal sodium transport may elicit activation of the brain RAS and stimulation of the central sympathetic nervous system, particularly under conditions of salt loading. High salt intake increased plasma sodium concentration both in normotensive Wistar rats and in SHRs [28]. As a consequence, sodium concentration increased in the cerebrospinal fluid (CSF) and in the extracellular fluid of the central nervous system (CNS) preceding an increase in BP and heart rate [29]. A similar response was also observed in Dahl S but not in Dahl R and WKY rats on high-salt diet [29]. The mechanisms mediating sodium transport across the blood-brain barrier are not yet fully elucidated. Sodium regulating mechanisms similar to those in the kidney, *i.e.* *via* aldosterone, mineralocorticoid receptors, epithelial sodium channels (ENaC) and Na/K-ATPase also exist in the brain [30], and recent findings have suggested that both ENaC and Na/K-ATPase regulate sodium transport in the choroid plexus [31]. 

*Endogenous ouabain (EO):* An important mediator of increased blood pressure under high salt intake is endogenous ouabain (EO). EO belongs to a group of endogenous cardiotonic steroids which exert digitalis-like effects. It is secreted in the brain from the supraoptic and median preoptic nuclei of the hypothalamus, and additionally from the adrenal cortex in humans and rats (reviewed in [32]). In SHRs, brain secretion is stimulated by elevated CSF sodium levels [5]. EO inhibits the activity of the Na/K-ATPase in the choroid plexus and hence might prevent a further increase in CSF sodium concentration. Additionally, it activates the brain RAS in the median preoptic nucleus and stimulates the release of angiotensin (ANG) II in SHRs and Dahl S rats [30]. Injections of the AT1 blocker losartan into the median preoptic nucleus prevented the pressor response to high CSF sodium, thus confirming the role of ANG II as a mediator in this reaction [27]. The brain RAS again contributes to reduction of the Na/K-ATPase activity [33,34]. Moreover, stimulation of AT1 elicits the release of marinobufagenin (MBG), another cardiotonic steroid from the adrenal cortex [35]. MBG induces a sustained inhibition of the α_1_ isoform of the Na/K-ATPase. Besides the brain, both EO and MBG mainly act on the kidney and on the vasculature where they mediate a long-term increase in BP [32,35]. 

*RAS:* Increased activity of the brain RAS is involved in the development and maintenance of hypertension and plays a central role in genetically hypertensive rat models such as SHRs or Dahl S rats. Long-term salt loading in young SHRs exaggerated the development of hypertension, and this effect has been considered to result from increased sodium concentration in the CSF and enhanced activities of the brain RAS and the SNS [25,36]. Overactivity of the brain RAS has been thought to result from increased renin [37], ANG II [38,39] or aldosterone levels [40,41] or from increased numbers of ANG II binding sites [42,43]. Additionally, increased sensitivity to ANG II supports the pressor effects of brain RAS stimulation [44,45]. The anterior hypothalamic area (AHA) is an important site of the brain RAS [46]. It is stimulated both by endogenous ANG II and by ICV hypertonic saline infusion, and sensitivity to these stimuli is much higher in SHRs and Dahl S rats than in WKY [45]. It has therefore been suggested that the brain RAS might be even more important for the development of salt sensitivity than for hypertension [16]. In salt-sensitive rat strains such as Dahl S rats, high salt intake increases the hypothalamic synthesis of aldosterone, presumably *via* elevated CSF sodium concentration. This steroid in turn stimulates mineralocorticoid receptors and thus activates central mechanisms contributing to salt-induced hypertension [40]. ICV infusion of aldosterone exerted effects similar to those of increased salt intake [47], and ICV application of mineralocorticoid or ENaC antagonists inhibited or stopped the development of hypertension [48,49]. Activation of the brain RAS induces a pressor response that is considered to be elicited through arginin-vasopressin dependent pathways or by increased sympathoexcitation and reduced sympathoinhibition [25,27,50]. Application of AT1 blockers or ACEI into the AHA or into CSF induced a depressor response in SHRs [36,46], while central aldosterone inhibition prevented sympathetic hyperactivity and hypertension in both Dahl S and Wistar rats [40,41]. 

*SNS*: Stimulation of EO and RAS in the brain elicits activation of the SNS [26], which plays a crucial role in the pressor effects of high salt intake in SHRs and Dahl S rats [26,29,51]. Both EO and RAS components are present in the hypothalamus and can mediate sympathoexcitatory and pressor responses to high salt intake in these rat strains [27,52,53,54]. EO increases the intracellular concentrations of both sodium and calcium *via* inhibition of Na/K-ATPase, thus enhancing neuronal excitability and causing central sympathetic activation [30]. 

Additionally, EO and ANG II can modulate the arterial baroreflex and thus contribute to sympathetic activation. While normotensive rats respond to high salt intake with sensitization of the baroreflex, enhanced EO activity in SHRs abolishes this sensitization or even results in desensitization leading to exaggeration of sympathetic activation [55,56]. Attenuation of baroreflex control by RAS components such as ANG II and aldosterone has been demonstrated in both animals and humans [57,58,59]. In SHRs with and without salt loading, we observed heart rate in control SHRs to be about 6% higher than in normotensive WKY, while heart rate in salt-loaded SHRs was 18% higher than in WKY. Treatment with the ACEI captopril reduced heart rate to control levels despite sustained salt-loading [11]. In humans, altered baroreflex sensitivity is associated with genetic polymorphism in aldosterone synthase indicating at least a partially hereditary basis for this baroreflex variation [60]. A recent study on hypertensive humans with different levels of salt sensitivity demonstrated decreasing baroreflex sensitivity to be associated with increasing salt sensitivity index [61]. ICV or hypothalamic application of either losartan or EO-binding antibodies or blockade of either sodium channels or mineralocorticoid receptors in the brain prevented all effects of sympathoexcitation in rats [5,26,27,62]. Finally, reduction of the endogenous NO production due to down-regulation of the inducible NO synthase (iNOS) at the medullary origin of sympathetic neurogenic vasomotor tone has been suggested to be a mechanism of sympathetic activation in SHRs [63].

Summarizing the brain mechanisms involved in salt-sensitive hypertension, enhanced activity of the brain RAS and increased sympathetic activity are the pivotal steps in this pathogenic process. Elevated CSF sodium concentration and EO release make a key contribution to this activation. Renal and vascular effects then mediate the increase in BP.

### 2.2. Kidney

Genetic or acquired impairments of renal sodium excretion have been proposed to underlie the development of hypertension. Cross-transplantation experiments in genetically hypertensive rat models [16,64,65] revealed that transplantation of a kidney from a normotensive animal into one from a hypertensive strain prevented the development of hypertension in the recipient; vice versa, kidney transplantation from hypertensive into normotensive rats induced hypertension. 

In both genetically hypertensive rats and humans, sodium retention is increased and excretion reduced compared to normotensive controls [66]. There are many possible causes for these differences. SHR strains and hypertensive patients may show characteristic structural differences to normotensive controls in the kidney such as a reduced number of glomeruli [67,68]. Congenital defects [69], environmental factors in the intrauterine or early postnatal period [70,71,72] or aquired renal injury such as tubulointerstitial renal disease [73,74] are also discussed as potential causative factors. 

Functional abnormities also contribute to sodium retention and hypervolemia, and thus to hypertension and salt sensitivity. Spontaneously hypertensive animals show sensitization of the tubuloglomerular feedback mechanism that leads to reduced glomerular filtration rate and urine excretion rate [75,76]. Renal sympathetic nerve activity (RSNA) regulates renin secretion and modulates renal vascular resistance, renal blood flow and glomerular filtration rate [77,78,89,80]. RSNA is elevated in EH patients [77,81] as well as in SHRs [82,83,84]. This is generally thought to result from hyperactivity of the SNS [47,82,85], from blunted sympathoinhib­i­tion after hyperosmolar central stimulation [80,84] or from impaired baroreflex control [55,56]. Renal denervation in hypertensive rat models delayed the development and blunted the severity of hypertension [86,87]. In newborn SHRs, sympathectomy led to a decrease in long-term arterial pressure, and this effect could be transferred into untreated SHRs by kidney transplantation [87]. 

Natriuresis can be impaired by abnormal function of the renal dopamine system, which normally inhibits the renal Na/K-ATPase. At high salt intake SHRs fail to respond to dopamine [88]. Reduced renal dopamine function is often associated with a predominating antinatriuretic function of the RAS both in genetically hypertensive rats and humans [22]. In normotensive rats, dopamine receptor subtypes decrease AT1 receptor expression, but this effect is lost in SHRs [89]. *Vice versa*, stimulation of AT1 receptors increases dopamine receptor type 1 in WKY but not in SHRs [90]. Dysfunction of the renal kallikrein-kinin system may also contribute to the development of salt-sensitive hypertension in animal models and humans [91,92].

*Natriuretic hormone:* Reduced natriuresis under conditions of high salt intake has often been proposed to reflect the function of a theoretical natriuretic hormone [66,93]. Initially, endogenous inhibitors of Na/K-ATPase similar to ouabain (EO) were considered as candidates for this natriuretic hormone. Natriuresis can be considered to be a compensatory mechanism reducing fluid volume in the body. However, inhibition of Na/K-ATPase in vessels induces vasoconstriction and thus, hypertension [94]. Furthermore, in the plasma of hypertensive patients, elevated concentrations of endogenous inhibitors of Na/K-ATPase have been found [94,95], while antagonists of EO have been demonstrated to exert potent antihypertensive effects in various hypertensive rat models and in patients [24,96]. A high-salt diet increased plasma EO concentration even in normotensive humans, the increase being more than 10-fold compared to normal salt intake, supporting the suggested role of circulating EO in the pathogenesis of salt-sensitive hypertension [97]. Peripheral EO is secreted from the adrenal cortex and inhibits the α_2_ type Na/K-ATPase [98,99,100] which is expressed in vascular smooth muscle cells (VSMC). In the kidney, however, EO interacts with the α_1_ type Na/K-ATPase in the caveolae, reduces its internalization and thus, increases the net activity [32]. In this way, EO increases sodium reabsorption in the renal proximal tubule [96] and does not exert a direct natriuretic effect [32]. This has been demonstrated in the Milan hypertensive rat strain (MHS) which has genetic polymorphism of the cytoskeletal protein α-adducin. The mutated α-adducin allele has been assumed to be the genetic basis for higher Na/K-ATPase activity leading to sodium and fluid retention both in rats and humans [101,102]. In a Belgian population study, polymorphisms of the α-adducin gene were accompanied by significantly elevated plasma EO concentration [103]. Acute saline infusion unmasked an interaction between α-adducin gene mutations and elevated plasma EO concentrations in hypertensive patients, probably mediated by augmented sensitivity of the vascular sodium pump [104] suggesting that these two mechanisms are associated with salt-sensitive forms of genetic hypertension in rats and humans [102,104].

Natriuresis is induced by another endogenous steroid from the adrenal cortex, the bufadienolide marinobufagenin (MBG). This is an inhibitor of the α_1_ type Na/K-ATPase, the main isoform in the kidney [93,105]. Through its action on both renal and vascular Na/K-ATPase MBG has natriuretic and vasoconstrictor effects [105,106,107]. High sodium intake induced an initial transient stimulation of EO and a subsequent progressive increase of MBG both in Dahl S rats and in humans [106,108]. In Dahl S rats, elevation in brain EO increased MBG secretion from the adrenal cortex, and this effect was blocked by the AT1 receptor antagonist losartan [35,109]. In elderly normotensive women on a high-salt diet, renal MBG excretion was correlated positively with urine sodium excretion and inversely with systolic blood pressure [108]. Hence, it has been suggested that the hypothetical natriuretic hormone referred to above might actually be a result of the combined effects of EO and MBG [93].

As mentioned above, these important mediators of genetic hypertension are also secreted in the hypothalamus and may contribute to hypertension independently of the primary renal defects. To date, brain mechanisms have been considered to be the main causative factors in genetic hypertension and salt-sensitivity [30,33].

*RAS:* In both human EH and in the SHR model, the function of circulating RAS is altered compared to normotensive controls. The kidney is the classic target organ of the circulating RAS. Variants of genes such as ACE, angiotensinogen or AT1 receptor modulate activity and expression not only of components of the brain RAS but also those of the systemic RAS. The importance of the kidney with respect to altered RAS function has been clearly shown in transplantation experiments in SHRs demonstrating that the hypotensive effects of treatment with ACEI can be transferred into a hypertensive recipient by kidney transplantation [110].

Normotensive animals respond to salt loading with down-regulation of the activity of the RAS [111]. This salt-induced down-regulation of the ANG II synthesis is attenuated [111] or even abolished in the SHR [112]. Hence, salt loading in SHRs increases ANG II production and renal ANG II stores [112], but not plasma aldosterone concentration [111]. Additionally, impaired degradation of ANG II may contribute to an elevated concentration of circulating ANG II [113]. However, the main effects of the RAS in the pathogenesis of salt-sensitive hypertension are exerted by local RAS activation causing exaggerated vasoconstriction as will be discussed below.

### 2.3. Vasculature and heart

Most of the mechanisms described above have an impact on the cardiovascular system, particularly on the vasculature as the most important target organ mediating hypertension. Elevated sympathetic activity, ANG II, endogenous Na/K-ATPase inhibitors and other influences exert their hypertensive effects mainly *via* vascular smooth muscles inducing vasoconstriction, and this reaction is more pronounced on high salt intake. Heart catheterization in six month old SHRs showed that mean aortic pressure (MAP) of SHRs under normal salt intake was 62% higher than in age-matched WKY, and total peripheral resistance (TPR) was mildly but not significantly increased [11]. In this study, salt loading (1% NaCl in drinking water) in SHRs did not further increase MAP, while TPR was almost doubled compared to WKY. Three different treatments, an ACEI (captopril), a β-adrenergic blocker (propranolol) or a calcium antagonist (verapamil), were compared in the salt-loaded SHRs. All reduced TPR but this decrease was most pronounced and only significant with captopril that completely abolished the effect of salt-loading [11]. 

*SNS:* Sympathetic innervation is the main source of the basal vasomotor tone. Sympathetic activity and vascular responsiveness are modified in genetic forms of hypertension and under conditions of excessive salt loading. SHRs show elevated sympathetic activity and increased concentrations of catecholamines (CA) in the serum and in various tissues [114,115]. Sympathectomy in neonatal SHRs completely prevented the development of hypertension [116]. Several mechanisms may be involved in this altered reaction. VSMC from rats fed a high-salt diet showed enhanced responses to noradrenaline [117]. ANG II or EO can activate the sympathetic nervous system and sensitize VSMC of SHRs to sympathetic innervation or to CA. EO is thought to induce this sensitization and to increase the activity of endothelial ACE and local ANG II synthesis [118,119]. ANG II is a known stimulator of norepinephrine release from peripheral sympathetic nerve terminals. On the contrary, NO reduces norepinephrine release from sympathetic nerve endings in the rat heart [120] and in the vasculature of rats and humans [121,122]. Correspondingly, in animal studies NOS inhibition has been shown to enhance the vasoconstrictor response to α-adrenergic agonists [123] and to eliminate the vasodilating effect of nitroxidergic innervation [124]. In hypertensive patients or rat models under conditions of increased salt intake, NO release by the vascular endothelium is reduced due to down-regulation or inhibition of NOS [63,125,126,127], thus increasing the sympathetic vasomotor tone [63]. NO production in the heart is also impaired, causing an enhanced inotropic response to β-adrenergic stimulation in patients with left ventricular dysfunction [128]. 

*EO and MBG:* Inhibitors of Na/K-ATPase such as EO and MBG exert similar effects on VSMC and induce vasoconstriction. Increased calcium influx and elevated cytosolic calcium concentration in VSMC are the main mediators of long-term elevated myogenic tone, vasoconstriction and hypertension in this process [32,66,100,129]. In healthy humans, ouabain administration increased peripheral vascular resistance by about 30%, and this effect was completely abolished by blockade of calcium influx by nifedipine [130]. The effect of the intracellular calcium concentration is exaggerated by ouabain-induced activation of sympathetic vasomotor innervation and sensitization of VSMC (as discussed above). EO secretion from the adrenal cortex is stimulated by ANG II *via* AT2 receptors [131]. ANG II is also an important link between EO and MBG release. In Dahl S rats, elevation in brain EO increased the MBG secretion from the adrenal cortex, and this effect was blocked by the AT1 receptor antagonist losartan [35,109].

*RAS:* Ouabain and ouabain-like substances are involved in local RAS activation [119]. Increased local generation of ANG II is thought to contribute to enhanced vasoconstriction and elevation of BP in patients with elevated circulating levels of EO and in rats with ouabain-induced hypertension [132]. VSMC of SHRs have a significantly greater production of ANG II compared to WKY [133]. ANG II can modulate endothelial function, particularly the endothelial release of vasoactive factors. The vascular endothelium regulates cardiovascular function by releasing a large number of vasoactive factors involving both dilating factors such as NO and endothelium-derived hyperpolarizing factor (EDHF) and constrictive factors such as endothelin (ET)-1 or ANG II. These modulators are well balanced under physiological conditions in humans, but in hypertension or under conditions of high salt intake, endothelial dysfunction may lead to impaired vasodilation [134]. Decreased bioavailability of NO plays a pivotal role in this endothelial dysfunction. NO deficiency is considered to prevent the usual down-regulatory effect of dietary sodium on ANG II synthesis in SHRs [111]. In addition to increased local ANG II production, the vascular reaction to ANG II is elevated in SHRs on a high-salt diet [7]. ANG II induces endothelial release of vasoconstrictors such as ET-1 and prostanoids. This effect was significantly greater in rats at high salt intake and in SHRs than in normotensive or low-salt diet controls [135,136,137,138]. Furthermore, ANG II impairs the release of EDHF and hence, relaxation to acetylcholine [139]. Finally, ANG II induces oxidative stress *via* stimulation of NAD(P)H oxidase and formation of reactive oxygen species (ROS) [140,141,142], which act as intracellular second messengers in signalling cascades leading to endothelial dysfunction, atherosclerotic changes, VSMC growth and remodelling of the extracellular matrix [143,144,145,146]. 

*Consequential cardiovascular damage:* A variety of humoral factors such as CA [116] and ANG II contribute to remodelling in the heart and vessels. ANG II can induce cardiovascular hypertrophy and remodelling in hypertensive patients as well as in animal models through different pathways such as increased ET-1 release [135], enhanced oxidative stress [146,147], increased mineralocorticoid receptor signals [147] or stimulation of sodium-proton exchange [148] and increase in free cytosolic calcium concentration [149]. In EH patients, a one-year treatment with either the β-blocker atenolol or the AT1 receptor blocker losartan reduced systolic BP by about 20 mmHg and diastolic BP by 15 mmHg. However, only losartan but not the β-blocker corrected structural changes and endothelial dysfunction in resistance arteries [150]. This finding demonstrates that the remodelling effects are at least partly independent of BP, and emphasizes the crucial role of ANG II in growth and remodelling of vessels. 

High salt intake can enhance the remodelling processes in various tissues. SHRs on a high-salt diet, compared to SHRs on a normal salt intake, developed structural changes in arteries associated with increased stiffness and hypertrophy [7]. In Wistar rats, salt loading attenuated the circulating RAS but not local RAS in the heart and kidneys, and led to a significant increase in relative left ventricular and kidney weight after four weeks [151]. In Sprague-Dawley rats, ANG II injection during high-salt diet exaggerated the salt-induced cardiac hypertrophy independently of BP, while ANG II administered on low-salt diet did not cause hypertrophy at similar BP levels [6]. In Dahl S rats, high-salt diet increased RAS activity in the CNS, heart and kidneys, and caused hypertension as well as cardiovascular and renal hypertrophy and fibrosis. RAS inhibition by AT1 receptor blockers or ACEI attenuated hypertension, completely prevented tissue fibrosis and partially prevented cardiac hypertrophy, indicating that local RAS in these tissues make a key contribution to salt-induced fibrosis [152,153]. In our own study, we compared the effects of treatment with the ACEI captopril and the calcium antagonist verapamil on BP and on cardiac remodelling in salt-loaded SHRs at 6 months of age [11]. Blood pressure was significantly higher in SHRs than in age-matched WKY but did not further increase under salt loading. Relative heart weight, however, was significantly higher than in SHRs at normal salt intake. Although neither captopril nor verapamil had an antihypertensive effect, they significantly reduced the mRNA expression of atrial natriuretic peptide, a marker of cardiac hypertrophy, and of collagen type I and III in the left ventricle [11]. These findings are confirmed by several studies reporting that ACEI therapy leads to regression of cardiac hypertrophy or vascular lesions in rats even in the absence of antihypertensive effects [154,155,156].

## 3. Therapeutic Effects of RAS Antagonists

The RAS, including the circulating as well as the various local elements, plays a pivotal role in the development of hypertension and particularly in the response to high salt intake. Consequently, antagonists of the RAS such as blockers of the AT1 receptor or inhibitors of ACE are potent antihypertensive drugs. In various animal models, the antihypertensive effect was demonstrated to result from a reduction in total peripheral resistance [157]. Moreover, ACEI can indirectly inhibit norepinephrine release from the sympathetic nerve terminals *via* reduced formation of vascular ANG II [158]. Studies comparing different antihypertensive therapies in rats demonstrate that ACEI or AT1 blockers have equally strong or even stronger effects on blood pressure than other lines of therapy involving blockers of adrenoceptors or calcium channels [11,154,156]. Additionally, RAS antagonists exert cardioprotective effects, *i.e.* antihypertrophic and antifibrotic effects on the heart and on vessels both in rats and humans [154,156,159]. However, it has been repeatedly reported that the efficiency of RAS antagonists, particularly of ACEI, is reduced under high salt intake in rats and in patients [7,36,160,161,162]. In some rat strains, such as Wistar rats or SHRs, high-salt diet suppresses the circulating RAS to increase sodium excretion as a compensation for the high sodium intake [36,151]. In contrast, salt-sensitive rat strains such as Dahl S rats responded with an initial suppression followed by marked activation of the circulating RAS [40]. Local RAS such as in the brain, heart, arteries and kidneys, however, are not reduced by high salt intake but are rather activated in SHRs, Wistar and Dahl S rats [36,40,151,152,153]. Hence, under conditions of high salt intake, higher doses of RAS antagonists might be necessary to block the activated tissue RAS, particularly the brain RAS [152]. ICV application of captopril exerted similar hypotensive effects in WKY and even greater effects in SHRs on a high compared to regular salt diet, although the depressor effects of intravenous captopril application in SHRs were reduced by salt loading [36]. In hypertensive patients, the antihypertensive effects of captopril can be enhanced when it is given in combination with a diuretic or after salt depletion [157]. Consequently, strict reduction of salt intake must be demanded to sufficiently decrease BP and to reverse consequential damage to organs such as heart and vessels.

## 4. Conclusions

The RAS occupies a central position in the pathogenesis of genetic forms of hypertension such as in SHRs or in EH patients. Since it can be activated under conditions of elevated salt consumption, it has particular importance for the development of salt sensitivity. In this respect, the brain RAS plays a pivotal role as it activates the central sympathetic nervous system. Moreover, the RAS exerts effects which synergize with those of other prohypertensive mechanisms on peripheral target organs, such as reduction of sodium excretion or vasoconstriction. Finally, the RAS induces structural changes leading to consequential target organ damage such as cardiac hypertrophy and fibrosis. Reducing the activity of the RAS by AT1 receptor blockade or ACE inhibition is a particularly important strategy of antihypertensive treatment, especially in salt-sensitive hypertension, as it decreases BP by several mechanisms and additionally, prevents consequential target organ damage. However, accompanying reduction of salt consumption is required to improve the therapeutic effects.

## References

[B1-pharmaceuticals-03-00940] Garcia E.A., Newhouse S., Caulfield M.J., Munroe P.B. (2003). Genes and Hypertension. Curr. Pharm. Des..

[B2-pharmaceuticals-03-00940] Weinberger M.H. (1996). Salt sensitivity of blood pressure in humans. Hypertension.

[B3-pharmaceuticals-03-00940] Frohlich E.D., Varagic J. (2005). Sodium directly impairs target organ function in hypertension. Curr. Opin. Cardiol..

[B4-pharmaceuticals-03-00940] Calhoun D.A., Zhu S., Wyss J.M., Oparil S. (1994). Diurnal blood pressure variation and dietary salt in spontaneously hypertensive rats. Hypertension.

[B5-pharmaceuticals-03-00940] Huang B.S., Leenen F.H. (2002). Brain amiloride-sensitive Phe-Met-Arg-Phe-NH(2)--gated Na(+) channels and Na(+)-induced sympathoexcitation and hypertension. Hypertension.

[B6-pharmaceuticals-03-00940] Morgan T.O., Aubert J.F., Wang Q. (1998). Sodium, angiotensin II, blood pressure and cardiac hypertrophy. Kidney Int..

[B7-pharmaceuticals-03-00940] Labat C., Lacolley P., Lajemi M., de Gasparo M., Safar M.E., Benetos A. (2001). Effects of valsartan on mechanical properties of the carotid artery in spontaneously hypertensive rats under high-salt diet. Hypertension.

[B8-pharmaceuticals-03-00940] Ahn J., Varagic J., Slama M., Susic D., Frohlich E.D. (2004). Cardiac structural and functional responses to salt loading in SHR. Am. J. Physiol. Heart Circ. Physiol..

[B9-pharmaceuticals-03-00940] Varagic J., Frohlich E.D., Díez J., Susic D., Ahn J., González A., López B. (2006). Myocardial fibrosis, impaired coronary hemodynamics, and biventricular dysfunction in salt-loaded SHR. Am. J. Physiol. Heart Circ. Physiol..

[B10-pharmaceuticals-03-00940] Sasaki Y., Fujimura M., Furukawa M., Kubo T. (2006). Sensitivity of pressor responses to central hypertonic saline is greatly enhanced even in pre-hypertensive spontaneously hypertensive rats. Neurosci. Lett..

[B11-pharmaceuticals-03-00940] Ziegelhöffer-Mihalovicova B., Arnold N., Marx G., Tannapfel A. (2006). Effects of salt loading and various therapies on cardiac hypertrophy and fibrosis in young spontaneously hypertensive rats. Life Sci..

[B12-pharmaceuticals-03-00940] Beeks E., Kessels A.G., Kroon A.A., van der Klauw M.M., de Leeuw P.W. (2004). Genetic predisposition to salt-sensitivity: a systematic review. J. Hypertens..

[B13-pharmaceuticals-03-00940] Iwai N., Tsujita Y., Kinoshita M. (1998). Isolation of a chromosome 1 region that contributes to high blood pressure and salt sensitivity. Hypertension.

[B14-pharmaceuticals-03-00940] Ely D., Turner M., Milsted A. (2000). Review of the Y chromosome and hypertension. Braz. J. Med. Biol. Res..

[B15-pharmaceuticals-03-00940] Aneas I., Rodrigues M.V., Pauletti B.A., Silva G.J., Carmona R., Cardoso L., Kwitek A.E., Jacob H.J., Soler J.M., Krieger J.E. (2009). Congenic strains provide evidence that four mapped loci in chromosomes 2, 4, and 16 influence hypertension in the SHR. Physiol. Genomics.

[B16-pharmaceuticals-03-00940] Johnson M.D., He L., Herman D., Wakimoto H., Wallace C.A., Zidek V., Mlejnek P., Musilova A., Simakova M., Vorlicek J., Kren V., Viklicky O., Qi N.R., Wang J., Seidman C.E. (2009). Dissection of chromosome 18 blood pressure and salt-sensitivity quantitative trait loci in the spontaneously hypertensive rat. Hypertension.

[B17-pharmaceuticals-03-00940] Johnson A.G., Nguyen T.V., Davis D. (2001). Blood pressure is linked to salt intake and modulated by the angiotensinogen gene in normotensive and hypertensive elderly subjects. J. Hypertens..

[B18-pharmaceuticals-03-00940] Hiraga H., Oshima T., Watanabe M., Ishida M., Ishida T., Shingu T., Kambe M., Matsuura H., Kajiyama G. (1996). Angiotensin I-converting enzyme gene polymorphism and salt sensitivity in essential hypertension. Hypertension.

[B19-pharmaceuticals-03-00940] Poch E., González D., Giner V., Bragulat E., Coca A., de La Sierra A. (2001). Molecular basis of salt sensitivity in human hypertension. Evaluation of renin-angiotensin-aldosterone system gene polymorphisms. Hypertension.

[B20-pharmaceuticals-03-00940] Shimoike H., Iwai N., Kinoshita M. (1997). Genetic analysis of renin gene expression in the central nervous system of spontaneously hypertensive rats. Neurosci. Lett..

[B21-pharmaceuticals-03-00940] Pamies-Andreu E., Ramirez-Lorca R., Stiefel García-Junco P., Muñiz-Grijalbo O., Vallejo-Maroto I., Garcia Morillo S., Miranda-Guisado M.L., Ortíz J.V., Carneado de la Fuente J. (2003). Renin-angiotensin-aldosterone system and G-protein beta-3 subunit gene polymorphisms in salt-sensitive essential hypertension. J. Hum. Hypertens..

[B22-pharmaceuticals-03-00940] Jose P.A., Eisner G.M., Felder R.A. (2003). Dopamine and the kidney: a role in hypertension?. Curr. Opin. Nephrol. Hypertens..

[B23-pharmaceuticals-03-00940] Cusi D., Barlassina C., Taglietti M.V. (2003). Genetics of human arterial hypertension. J. Nephrol..

[B24-pharmaceuticals-03-00940] Manunta P., Ferrandi M., Messaggio E., Ferrari P. (2006). A new antihypertensive agent that antagonizes the prohypertensive effect of endogenous ouabain and adducin. Cardiovasc. Hematol. Agents Med. Chem..

[B25-pharmaceuticals-03-00940] Takata Y., Yamashita Y., Takishita S., Tsuchihashi T., Tomita Y., Kimura Y., Koga T., Fujishima M. (1988). Central and peripheral mechanisms of the enhanced hypertension following long-term salt loading in spontaneously hypertensive rats. *Jpn*. Circ. J..

[B26-pharmaceuticals-03-00940] Huang B.S., Leenen F.H. (1996). Brain "ouabain" and angiotensin II in salt-sensitive hypertension in spontaneously hypertensive rats. Hypertension.

[B27-pharmaceuticals-03-00940] Budzikowski A.S., Leenen F.H. (2001). ANG II in median preoptic nucleus and pressor responses to CSF sodium and high sodium intake in SHR. Am. J. Physiol. Heart Circ. Physiol..

[B28-pharmaceuticals-03-00940] Fang Z., Carlson S.H., Peng N., Wyss J.M. (2000). Circadian rhythm of plasma sodium is disrupted in spontaneously hypertensive rats fed a high-NaCl diet. Am. J. Physiol. Regul. Integr. Comp. Physiol..

[B29-pharmaceuticals-03-00940] Huang B.S., van Vliet B.N., Leenen F.H. (2004). Increases in CSF [Na+] precede the increases in blood pressure in Dahl S rats and SHR on a high-salt diet. Am. J. Physiol. Heart Circ. Physiol..

[B30-pharmaceuticals-03-00940] Huang B.S., Amin M.S., Leenen F.H. (2006). The central role of the brain in salt-sensitive hypertension. Curr. Opin. Cardiol..

[B31-pharmaceuticals-03-00940] Amin M.S., Reza E., Wang H., Leenen F.H. (2009). Sodium transport in the choroid plexus and salt-sensitive hypertension. Hypertension.

[B32-pharmaceuticals-03-00940] Bagrov A.Y., Shapiro J.I., Fedorova O.V. (2009). Endogenous cardiotonic steroids: Physiology, pharmacology, and novel therapeutic targets. Pharmacol. Rev..

[B33-pharmaceuticals-03-00940] van Huysse J.W. (2007). Endogenous brain Na pumps, brain ouabain-like substance and the alpha2 isoform in salt-dependent hypertension. Pathophysiology.

[B34-pharmaceuticals-03-00940] van Huysse J.W., Hou X. (2004). Pressor response to CSF sodium in mice: mediation by a ouabain-like substance and renin-angiotensin system in the brain. Brain Res..

[B35-pharmaceuticals-03-00940] Fedorova O.V., Agalakova N.I., Talan M.I., Lakatta E.G., Bagrov A.Y. (2005). Brain ouabain stimulates peripheral marinobufagenin *via* angiotensin II signalling in NaCl-loaded Dahl-S rats. J. Hypertens..

[B36-pharmaceuticals-03-00940] Takata Y., Yamashita Y., Takishita S., Kimura Y., Fujishima M. (1986). Brain renin angiotensin system contributes to the salt-induced enhancement of hypertension in SHR. Clin. Exp. Hypertens. A..

[B37-pharmaceuticals-03-00940] Schelling P., Meyer D., Loos H.E., Speck G., Phillips M.I., Johnson A.K., Ganten D. (1982). A micromethod for the measurement of renin in brain nuclei: its application in spontaneously hypertensive rats. Neuropharmacology.

[B38-pharmaceuticals-03-00940] Ganten D., Hermann K., Bayer C., Unger T., Lang R.E. (1983). Angiotensin synthesis in the brain and increased turnover in hypertensive rats. Science.

[B39-pharmaceuticals-03-00940] Phillips M.I., Kimura B. (1986). Converting enzyme inhibitors and brain angiotensin. J. Cardiovasc. Pharmacol..

[B40-pharmaceuticals-03-00940] Huang B.S., White R.A., Jeng A.Y., Leenen F.H. (2009). Role of central nervous system aldosterone synthase and mineralocorticoid receptors in salt-induced hypertension in Dahl salt-sensitive rats. Am. J. Physiol. Regul. Integr. Comp. Physiol..

[B41-pharmaceuticals-03-00940] Huang B.S., White R.A., Ahmad M., Jeng A.Y., Leenen F.H. (2008). Central infusion of aldosterone synthase inhibitor prevents sympathetic hyperactivity and hypertension by central Na^+^ in Wistar rats. Am. J. Physiol. Regul. Integr. Comp. Physiol..

[B42-pharmaceuticals-03-00940] Gehlert D.R., Speth R.C., Wamsley J.K. (1986). Quantitative autoradiography of angiotensin II receptors in the SHR brain. Peptides.

[B43-pharmaceuticals-03-00940] Gutkind J.S., Kurihara M., Castren E., Saavedra J.M. (1988). Increased concentration of angiotensin II binding sites in selected brain areas of spontaneously hypertensive rats. J. Hypertens..

[B44-pharmaceuticals-03-00940] Wright J.W., Sullivan M.J., Quirk W.S., Batt C.M., Harding J.W. (1986). Heightened pressor effect and dipsogenicity to intracerebroventricularly applied angiotensin II and III in spontaneously hypertensive rats. J. Hypertens. Suppl..

[B45-pharmaceuticals-03-00940] Kubo T., Hagiwara Y. (2006). Angiotensin II sensitivity of anterior hypothalamic area neurons is enhanced in both spontaneously hypertensive rats and Dahl salt-sensitive rats. Neurosci. Lett..

[B46-pharmaceuticals-03-00940] Kubo T., Yamaguchi H., Tsujimura M., Hagiwara Y., Fukumori R. (2000). An angiotensin system in the anterior hypothalamic area anterior is involved in the maintenance of hypertension in spontaneously hypertensive rats. Brain Res. Bull..

[B47-pharmaceuticals-03-00940] Huang B.S., Wang H., Leenen F.H. (2005). Chronic central infusion of aldosterone leads to sympathetic hyperreactivity and hypertension in Dahl S but not Dahl R rats. Am. J. Physiol. Heart Circ. Physiol..

[B48-pharmaceuticals-03-00940] Gomez-Sanchez E.P., Fort C., Thwaites D. (1992). Central mineralocorticoid receptor antagonism blocks hypertension in Dahl S/JR rats. Am. J. Physiol. Endocrinol. Metab..

[B49-pharmaceuticals-03-00940] Gomez-Sanchez E.P., Gomez-Sanchez C.E. (1995). Effect of central infusion of benzamil on Dahl S rat hypertension. Am. J. Physiol. Heart Circ. Physiol..

[B50-pharmaceuticals-03-00940] Lon S., Szczepanska-Sadowska E., Szczypaczewska M. (1996). Evidence that centrally released arginine vasopressin is involved in central pressor action of angiotensin II. Am. J. Physiol. Heart Circ. Physiol..

[B51-pharmaceuticals-03-00940] Wyss J.M., Mozaffari M.S., Roysommuti S. (1995). Contribution of the sympathetic nervous system to salt-sensitivity in lifetime captopril-treated spontaneously hypertensive rats. J. Hypertens..

[B52-pharmaceuticals-03-00940] Budzikowski A.S., Leenen F.H. (1997). Brain 'ouabain' in the median preoptic nucleus mediates sodium-sensitive hypertension in spontaneously hypertensive rats. Hypertension.

[B53-pharmaceuticals-03-00940] Budzikowski A.S., Huang B.S., Leenen F.H. (1998). Brain "ouabain", a neurosteroid, mediates sympathetic hyperactivity in salt-sensitive hypertension. Clin. Exp. Hypertens..

[B54-pharmaceuticals-03-00940] van Huysse J.W., Leenen F.H. (1998). Role of endogenous brain "ouabain" in the sympathoexcitatory and pressor effects of sodium. Clin. Exp. Hypertens..

[B55-pharmaceuticals-03-00940] Ono A., Kuwaki T., Kumada M., Fujita T. (1997). Differential central modulation of the baroreflex by salt loading in normotensive and spontaneously hypertensive rats. Hypertension.

[B56-pharmaceuticals-03-00940] Huang B.S., Leenen F.H. (1995). Brain 'ouabain,' sodium, and arterial baroreflex in spontaneously hypertensive rats. Hypertension.

[B57-pharmaceuticals-03-00940] Wang W. (1994). Chronic administration of aldosterone depresses baroreceptor reflex function in the dog. Hypertension.

[B58-pharmaceuticals-03-00940] Yee K.M., Struthers A.D. (1998). Endogenous angiotensin II and baroreceptor dysfunction: a comparative study of losartan and enalapril in man. Br. J. Clin. Pharmacol..

[B59-pharmaceuticals-03-00940] McMullan S., Goodchild A.K., Pilowsky P.M. (2007). Circulating angiotensin II attenuates the sympathetic baroreflex by reducing the barosensitivity of medullary cardiovascular neurones in the rat. J. Physiol..

[B60-pharmaceuticals-03-00940] Ylitalo A., Airaksinen K.E., Hautanen A., Kupari M., Carson M., Virolainen J., Savolainen M., Kauma H., Kesäniemi Y.A., White P.C., Huikuri H.V. (2000). Baroreflex sensitivity and variants of the renin angiotensin system genes. J. Am. Coll. Cardiol..

[B61-pharmaceuticals-03-00940] Coruzzi P., Parati G., Brambilla L., Brambilla V., Gualerzi M., Novarini A., Castiglioni P., di Rienzo M. (2005). Effects of salt sensitivity on neural cardiovascular regulation in essential hypertension. Hypertension.

[B62-pharmaceuticals-03-00940] Huang B.S., Leenen F.H. (2005). Blockade of brain mineralocorticoid receptors or Na^+^ channels prevents sympathetic hyperactivity and improves cardiac function in rats post-MI. Am. J. Physiol. Heart Circ. Physiol..

[B63-pharmaceuticals-03-00940] Chan J.Y., Wang L.L., Wu K.L., Chan S.H. (2001). Reduced functional expression and molecular synthesis of inducible nitric oxide synthase in rostral ventrolateral medulla of spontaneously hypertensive rats. Circulation.

[B64-pharmaceuticals-03-00940] Dahl L.K., Heine M. (1975). Primary role of renal homografts in setting chronic blood pressure levels in rats. Circ. Res..

[B65-pharmaceuticals-03-00940] Kawabe K., Watanabe T.X., Shiono K., Sokabe H. (1978). Influence on blood pressure of renal isografts between spontaneously hypertensive and normotensive rats, utilizing the F1 hybrids. Jpn. Heart J..

[B66-pharmaceuticals-03-00940] de Wardener H.E., Mac Gregor G.A. (1982). The natriuretic hormone and essential hypertension. The Lancet.

[B67-pharmaceuticals-03-00940] Keller G., Zimmer G., Mall G., Ritz E., Amann K. (2003). Nephron number in patients with primary hypertension. N. Engl. J. Med..

[B68-pharmaceuticals-03-00940] Skov K., Nyengaard J.R., Korsgaard N., Mulvany M.J. (1994). Number and size of renal glomeruli in spontaneously hypertensive rats. J. Hypertens..

[B69-pharmaceuticals-03-00940] Fogelgren B., Yang S., Sharp I.C., Huckstep O.J., Ma W., Somponpun S.J., Carlson E.C., Uyehara C.F.T., Lozanoff S. (2009). Deficiency in Six2 during prenatal development is associated with reduced nephron number, chronic renal failure, and hypertension in Br/+ adult mice. Am. J. Physiol. Renal Physiol..

[B70-pharmaceuticals-03-00940] Barker D.J., Osmond C., Golding J., Kuh D., Wadsworth M.E. (1989). Growth in utero, blood pressure in childhood and adult life, and mortality from cardiovascular disease. BMJ.

[B71-pharmaceuticals-03-00940] Zandi-Nejad K., Luyckx V.A., Brenner B.M. (2006). Adult Hypertension and Kidney Disease. The Role of Fetal Programming. Hypertension.

[B72-pharmaceuticals-03-00940] Marin E.C., Balbi A.P., Francescato H.D., Alves da Silva C.G., Costa R.S., Coimbra T.M. (2008). Renal structure and function evaluation of rats from dams that received increased sodium intake during pregnancy and lactation submitted or not to 5/6 nephrectomy. Ren. Fail..

[B73-pharmaceuticals-03-00940] Johnson R.J., Schreiner G.F. (1997). Hypothesis: the role of acquired tubulointerstitial disease in the pathogenesis of salt-dependent hypertension. Kidney Int..

[B74-pharmaceuticals-03-00940] Ray P.E., Suga S., Liu X.H., Huang X., Johnson R.J. (2001). Chronic potassium depletion induces renal injury, salt sensitivity, and hypertension in young rats. Kidney Int..

[B75-pharmaceuticals-03-00940] Kurokawa K. (1995). Role of the kidney in the genesis of hypertension: aberrant renal response to excess salt intake. Clin. Exp. Pharmacol. Physiol..

[B76-pharmaceuticals-03-00940] Persson A.E., Gutierrez A., Pittner J., Ring A., Ollerstam A., Brown R., Liu R., Thorup C. (2000). Renal NO production and the development of hypertension. Acta Physiol. Scand..

[B77-pharmaceuticals-03-00940] Oparil S. (1986). The sympathetic nervous system in clinical and experimental hypertension. Kidney Int..

[B78-pharmaceuticals-03-00940] Sato Y., Ando K., Ogata E., Fujita T. (1991). High-potassium diet attenuates salt-induced acceleration of hypertension in SHR. Am. J. Physiol..

[B79-pharmaceuticals-03-00940] Wyss J.M., Oparil S., Sripairojthikoon W. (1992). Neuronal control of the kidney: contribution to hypertension. Can. J. Physiol. Pharmacol..

[B80-pharmaceuticals-03-00940] Pedrino G.R., Rosa D.A., Korim W.S., Cravo S.L. (2008). Renal sympathoinhibition induced by hypernatremia: involvement of A1 noradrenergic neurons. Auton. Neurosci..

[B81-pharmaceuticals-03-00940] Esler M., Lambert G., Brunner-La Rocca H.P., Vaddadi G., Kaye D. (2003). Sympathetic nerve activity and neurotransmitter release in humans: translation from pathophysiology into clinical practice. Acta Physiol. Scand..

[B82-pharmaceuticals-03-00940] Judy W.V., Farrell S.K. (1979). Arterial baroreceptor reflex control of sympathetic nerve activity in the spontaneously hypertensive rat. Hypertension.

[B83-pharmaceuticals-03-00940] Caplea A., Seachrist D., Daneshvar H., Dunphy G., Ely D. (2002). Noradrenergic content and turnover rate in kidney and heart shows gender and strain differences. J. Appl. Physiol..

[B84-pharmaceuticals-03-00940] Guadagnini D., Gontijo J.A. (2006). Altered renal sodium handling in spontaneously hypertensive rats (SHR) after hypertonic saline intracerebroventricular injection: role of renal nerves. Life Sci..

[B85-pharmaceuticals-03-00940] Huang B.S., Ahmad M., Deng A.Y., Leenen F.H. (2007). Neuronal responsiveness to central Na^+^ in 2 congenic strains of Dahl salt-sensitive rats. Hypertension.

[B86-pharmaceuticals-03-00940] Greenberg S., Osborn J.L. (1994). Relationship between sodium balance and renal innervation during hypertension development in the spontaneously hypertensive rat. J. Hypertens..

[B87-pharmaceuticals-03-00940] Grisk O., Rose H.-J., Lorenz G., Rettig R. (2002). Sympathetic–renal interaction in chronic arterial pressure control. Am. J. Physiol. Regul. Integr. Comp. Physiol..

[B88-pharmaceuticals-03-00940] Lucas-Teixeira V.A., Vieira-Coelho M.A., Serrão P., Pestana M., Soares-da-Silva P. (2000). Salt intake and sensitivity of intestinal and renal Na^+^-K^+^ ATPase to inhibition by dopamine in spontaneous hypertensive and Wistar-Kyoto rats. Clin. Exp. Hypertens..

[B89-pharmaceuticals-03-00940] Zeng C., Luo Y., Asico L.D., Hopfer U., Eisner G.M., Felder R.A., Jose P.A. (2003). Perturbation of D1 dopamine and AT1 receptor interaction in spontaneously hypertensive rats. Hypertension.

[B90-pharmaceuticals-03-00940] Zeng C., Wang Z., Hopfer U., Asico L.D., Eisner G.M., Felder R.A., Jose P.A. (2005). Rat strain effects of AT1 receptor activation on D1 dopamine receptors in immortalized renal proximal tubule cells. Hypertension.

[B91-pharmaceuticals-03-00940] Iimura O., Shimamoto K. (1993). Salt and hypertension: water-sodium handling in essential hypertension. Ann. N. Y. Acad. Sci..

[B92-pharmaceuticals-03-00940] Katori M., Majima M. (2003). The renal kallikrein-kinin system: its role as a safety valve for excess sodium intake, and its attenuation as a possible etiologic factor in salt-sensitive hypertension. Crit. Rev. Clin. Lab. Sci..

[B93-pharmaceuticals-03-00940] Bagrov A.Y., Fedorova O.V. (2005). Cardenolide and bufadienolide ligands of the sodium pump. How they work together in NaCl sensitive hypertension. Front. Biosci..

[B94-pharmaceuticals-03-00940] Blaustein M.P., Hamlyn J.M. (1983). Role of a natriuretic factor in essential hypertension: an hypothesis. Ann. Intern. Med..

[B95-pharmaceuticals-03-00940] de Wardener H.E., Mac Gregor G.A. (1983). The relation of the natriuretic hormone to essential hypertension. Postgrad. Med. J..

[B96-pharmaceuticals-03-00940] Ferrari P., Ferrandi M., Valentini G., Manunta P., Bianchi G. (2007). Targeting Ouabain- and Adducin-dependent mechanisms of hypertension and cardiovascular remodeling as a novel pharmacological approach. Med. Hypotheses.

[B97-pharmaceuticals-03-00940] Manunta P., Hamilton B.P., Hamlyn J.M. (2006). Salt intake and depletion increase circulating levels of endogenous ouabain in normal men. Am. J. Physiol. Regul. Integr. Comp. Physiol..

[B98-pharmaceuticals-03-00940] Dostanic I., Paul R.J., Lorenz J.N., Theriault S., van Huysse J.W., Lingrel J.B. (2005). The alpha2-isoform of Na-K-ATPase mediates ouabain-induced hypertension 
in mice and increased vascular contractility *in vitro*. Am. J. Physiol. Heart Circ. Physiol..

[B99-pharmaceuticals-03-00940] Zhang J., Lee M.Y., Cavalli M., Chen L., Berra-Romani R., Balke C.W., Bianchi G., Ferrari P., Hamlyn J.M., Iwamoto T., Lingrel J.B., Matteson D.R., Wier W.G., Blaustein M.P. (2005). Sodium pump alpha2 subunits control myogenic tone and blood pressure in mice. J. Physiol..

[B100-pharmaceuticals-03-00940] Blaustein M.P., Zhang J., Chen L., Song H., Raina H., Kinsey S.P., Izuka M., Iwamoto T., Kotlikoff M.I., Lingrel J.B., Philipson K.D., Wier W.G., Hamlyn J.M. (2009). The pump, the exchanger, and endogenous ouabain: signaling mechanisms that link salt retention to hypertension. Hypertension.

[B101-pharmaceuticals-03-00940] Cusi D., Barlassina C., Azzani T., Casari G., Citterio L., Devoto M., Glorioso N., Lanzani C., Manunta P., Righetti M., Rivera R., Stella P., Troffa C., Zagato L., Bianchi G. (1997). Polymorphisms of alpha-adducin and salt sensitivity in patients with essential hypertension. Lancet.

[B102-pharmaceuticals-03-00940] Ferrandi M., Salardi S., Tripodi G., Barassi P., Rivera R., Manunta P., Goldshleger R., Ferrari P., Bianchi G., Karlish S.J.D. (1999). Evidence for an interaction between adducin and Na^+^-K^+^-ATPase: relation to genetic hypertension. Am. J. Physiol. Heart Circ. Physiol..

[B103-pharmaceuticals-03-00940] Wang J.G., Staessen J.A., Messaggio E., Nawrot T., Fagard R., Hamlyn J.M., Bianchi G., Manunta P. (2003). Salt, endogenous ouabain and blood pressure interactions in the general population. J. Hypertens..

[B104-pharmaceuticals-03-00940] Manunta P., Maillard M., Tantardini C., Simonini M., Lanzani C., Citterio L., Stella P., Casamassima N., Burnier M., Hamlyn J.M., Bianchi G. (2008). Relationships among endogenous ouabain, alpha-adducin polymorphisms and renal sodium handling in primary hypertension. J. Hypertens..

[B105-pharmaceuticals-03-00940] Fedorova O.V., Kolodkin N.I., Agalakova N.I., Lakatta E.G., Bagrov A.Y. (2001). Marinobufagenin, an endogenous alpha-1 sodium pump ligand, in hypertensive Dahl salt-sensitive rats. Hypertension.

[B106-pharmaceuticals-03-00940] Fedorova O.V., Talan M.I., Agalakova N.I., Lakatta E.G., Bagrov A.Y. (2002). Endogenous ligand of alpha(1) sodium pump, marinobufagenin, is a novel mediator of sodium chloride--dependent hypertension. Circulation.

[B107-pharmaceuticals-03-00940] Bagrov A.Y., Shapiro J.I. (2008). Endogenous digitalis: pathophysiologic roles and therapeutic applications. Nat. Clin. Pract. Nephrol..

[B108-pharmaceuticals-03-00940] Anderson D.E., Fedorova O.V., Morrell C.H., Longo D.L., Kashkin V.A., Metzler J.D., Bagrov A.Y., Lakatta E.G. (2008). Endogenous sodium pump inhibitors and age-associated increases in salt sensitivity of blood pressure in normotensives. Am. J. Physiol. Regul. Integr. Comp. Physiol..

[B109-pharmaceuticals-03-00940] Fedorova O.V., Zhuravin I.A., Agalakova N.I., Yamova L.A., Talan M.I., Lakatta E.G., Bagrov A.Y. (2007). Intrahippocampal microinjection of an exquisitely low dose of ouabain mimics NaCl loading and stimulates a bufadienolide Na/K-ATPase inhibitor. J. Hypertens..

[B110-pharmaceuticals-03-00940] Smallegange C., Kline R.L., Adams M.A. (2003). Transplantation of enalapril-treated kidneys confers persistent lowering of arterial pressure in SHR. Hypertension.

[B111-pharmaceuticals-03-00940] Hodge G., Ye V.Z., Duggan K.A. (2002). Dysregulation of angiotensin II synthesis is associated with salt sensitivity in the spontaneous hypertensive rat. Acta Physiol. Scand..

[B112-pharmaceuticals-03-00940] Meng Q.C., Durand J., Chen Y.F., Oparil S. (1995). Effects of dietary salt on angiotensin peptides in kidney. J. Am. Soc. Nephrol..

[B113-pharmaceuticals-03-00940] Mizutani S., Ishii M., Hattori A., Nomura S., Numaguchi Y., Tsujimoto M., Kobayshi H., Murohara T., Wright J. W. (2008). New insights into the importance of aminopeptidase A in hypertension. Heart Fail. Rev..

[B114-pharmaceuticals-03-00940] Pak C.H. (1981). Plasma adrenaline and noradrenaline concentrations of the spontaneously hypertensive rat. Jpn. Heart J..

[B115-pharmaceuticals-03-00940] Tsunoda M., Takezawa K., Santa T., Ina Y., Nagashima K., Ohmori K., Kobayashi S., Imai K. (2000). New approach for measurement of sympathetic nervous abnormality in conscious, spontaneously hypertensive rats. Jpn. J. Pharmacol..

[B116-pharmaceuticals-03-00940] Lee R.M., Triggle C.R., Cheung D.W., Coughlin M.D. (1987). Structural and functional consequence of neonatal sympathectomy on the blood vessels of spontaneously hypertensive rats. Hypertension.

[B117-pharmaceuticals-03-00940] Sofola O.A., Knill A., Hainsworth R., Drinkhill M. (2002). Change in endothelial function in mesenteric arteries of Sprague-Dawley rats fed a high salt diet. J. Physiol..

[B118-pharmaceuticals-03-00940] Rossoni L.V., Cunha V., Franca A., Vassallo D.V. (1999). The influence of nanomolar ouabain on vascular pressor responses is modulated by the endothelium. J. Cardiovasc. Pharmacol..

[B119-pharmaceuticals-03-00940] Padilha A.S., Rossoni L.V., Xavier F.E., Vassallo D.V. (2004). Ouabain at nanomolar concentration promotes synthesis and release of angiotensin II from the endothelium of the tail vascular bed of spontaneously hypertensive rats. J. Cardiovasc. Pharmacol..

[B120-pharmaceuticals-03-00940] Schwarz P., Diem R., Dun N.J., Förstermann U. (1995). Endogenous and exogenous nitric oxide inhibits norepinephrine release from rat heart sympathetic nerves. Circ. Res..

[B121-pharmaceuticals-03-00940] Vo P.A., Reid J.J., Rand M.J. (1992). Attenuation of vasoconstriction by endogenous nitric oxide in rat caudal artery. Br. J. Pharmacol..

[B122-pharmaceuticals-03-00940] Costa F., Christensen N.J., Farley G., Biaggioni I. (2001). NO modulates norepinephrine release in human skeletal muscle: implications for neural preconditioning. Am. J. Physiol. Regul. Integr. Comp. Physiol..

[B123-pharmaceuticals-03-00940] Patil R.D., di Carlo S.E., Collins H.L. (1993). Acute exercise enhances nitric oxide modulation of vascular response to phenylephrine. Am. J. Physiol..

[B124-pharmaceuticals-03-00940] Toda N., Kitamura Y., Okamura T. (1993). Neural mechanism of hypertension by nitric oxide synthase inhibitor in dogs. Hypertension.

[B125-pharmaceuticals-03-00940] Ni Z., Oveisi F., Vaziri N.D. (1999). Nitric Oxide Synthase Isotype Expression in Salt-Sensitive and Salt-Resistant Dahl Rats. Hypertension.

[B126-pharmaceuticals-03-00940] Fujiwara N., Osanai T., Kamada T., Katoh T., Takahashi K., Okumura K. (2000). Study on the Relationship Between Plasma Nitrite and Nitrate Level and Salt Sensitivity in Human Hypertension. Modulation of Nitric Oxide Synthesis by Salt Intake. Circulation.

[B127-pharmaceuticals-03-00940] Iaccarino G., Ciccarelli M., Sorriento D., Cipolletta E., Cerullo V., Iovino G.L., Paudice A., Elia A., Santulli G., Campanile A., Arcucci O., Pastore L., Salvatore F., Condorelli G., Trimarco B. (2004). AKT participates in endothelial dysfunction in hypertension. Circulation.

[B128-pharmaceuticals-03-00940] Hare J.M., Loh E., Creager M.A., Colucci W.S. (1995). Nitric oxide inhibits the positive inotropic response to beta-adrenergic stimulation in humans with left ventricular dysfunction. Circulation.

[B129-pharmaceuticals-03-00940] Iwamoto T., Kita S., Zhang J., Blaustein M.P., Arai Y., Yoshida S., Wakimoto K., Komuro I., Katsuragi T. (2004). Salt-sensitive hypertension is triggered by Ca^2+^ entry *via* Na^+^/Ca^2+^ exchanger type-1 in vascular smooth muscle. Nat. Med..

[B130-pharmaceuticals-03-00940] Kramer H.J., Glanzer K., Sorger M. (1985). The role of endogenous inhibition of Na-K-ATPase in human hypertension--sodium pump activity as a determinant of peripheral vascular resistance. Clin. Exp. Hypertens. A.

[B131-pharmaceuticals-03-00940] Laredo J., Shah J.R., Lu Z., Hamilton B.P., Hamlyn J.M. (1997). Angiotensin II Stimulates Secretion of Endogenous Ouabain From Bovine Adrenocortical Cells *via* Angiotensin Type 2 Receptors. Hypertension.

[B132-pharmaceuticals-03-00940] Hamlyn J.M., Hamilton B.P., Manunta P. (1996). Endogenous ouabain, sodium balance and blood pressure: a review and a hypothesis. J. Hypertens..

[B133-pharmaceuticals-03-00940] Hu W.Y., Fukuda N., Kanmatsuse K. (2002). Growth characteristics, angiotensin II generation, and microarray-determined gene expression in vascular smooth muscle cells from young spontaneously hypertensive rats. J. Hypertens..

[B134-pharmaceuticals-03-00940] Puddu P., Puddu G.M., Zaca F., Muscari A. (2000). Endothelial dysfunction in hypertension. Acta Cardiol..

[B135-pharmaceuticals-03-00940] Moreau P., d'Uscio L.V., Shaw S., Takase H., Barton M., Lüscher T.F. (1997). Angiotensin II increases tissue endothelin and induces vascular hypertrophy - Reversal by ETA-receptor antagonist. Circulation.

[B136-pharmaceuticals-03-00940] Ortiz M.C., Manriquez M.C., Romero J.C., Juncos L.A. (2001). Antioxidants block angiotensin II-induced increases in blood pressure and endothelin. Hypertension.

[B137-pharmaceuticals-03-00940] Zerrouk A., Auguet M., Chabrier P.E. (1998). Augmented endothelium-dependent contraction to angiotensin II in the SHR aorta: Role of an inducible cyclooxygenase metabolite. J. Cardiovasc. Pharmacol..

[B138-pharmaceuticals-03-00940] Sasser J.M., Pollock J.S., Pollock D.M. (2002). Renal endothelin in chronic angiotensin II hypertension. Am. J. Physiol. Regul. Integr. Comp. Physiol..

[B139-pharmaceuticals-03-00940] Goto K., Fujii K., Onaka U., Abe I., Fujishima M. (2000). Renin-Angiotensin System Blockade Improves Endothelial Dysfunction in Hypertension. Hypertension.

[B140-pharmaceuticals-03-00940] Cai H., Li Z., Dikalov S., Holland S.M., Hwang J., Jo H., Dudley Jr. S.C., Harrison D.G. (2002). NAD(P)H oxidase-derived hydrogen peroxide mediates endothelial nitric oxide production in response to angiotensin II. J. Biol. Chem..

[B141-pharmaceuticals-03-00940] Landmesser U., Cai H., Dikalov S., McCann L., Hwang J., Jo H., Holland S.M., Harrison D.G. (2002). Role of p47(phox) in vascular oxidative stress and hypertension caused by angiotensin II. Hypertension.

[B142-pharmaceuticals-03-00940] Mollnau H., Wendt M., Szöcs K., Lassègue B., Schulz E., Oelze M., Li H., Bodenschatz M., August M., Kleschyov A.L., Tsilimingas N., Walter U., Förstermann U., Meinertz T., Griendling K., Münzel T. (2002). Effects of angiotensin II infusion on the expression and function of NAD(P)H oxidase and components of nitric oxide/cGMP signaling. Circ. Res..

[B143-pharmaceuticals-03-00940] Touyz R.M., Schiffrin E.L. (2001). Increased generation of superoxide by angiotensin II in smooth muscle cells from resistance arteries of hypertensive patients: role of phospholipase D-dependent NAD(P)H oxidase-sensitive pathways. J. Hypertens..

[B144-pharmaceuticals-03-00940] Rueckschloss U., Quinn M.T., Holtz J., Morawietz H. (2002). Dose-dependent regulation of NAD(P)H oxidase expression by angiotensin II in human endothelial cells: protective effect of angiotensin II type 1 receptor blockade in patients with coronary artery disease. Arterioscler. Thromb. Vasc. Biol..

[B145-pharmaceuticals-03-00940] Lassègue B., Clempus R.E. (2003). Vascular NAD(P)H oxidases: specific features, expression, and regulation. Am. J. Physiol. Regul. Integr. Comp. Physiol..

[B146-pharmaceuticals-03-00940] Touyz R.M., Tabet F., Schiffrin E.L. (2003). Redox-dependent signalling by angiotensin II and vascular remodelling in hypertension. Clin. Exp. Pharmacol. Physiol..

[B147-pharmaceuticals-03-00940] Matsui H., Ando K., Kawarazaki H., Nagae A., Fujita M., Shimosawa T., Nagase M., Fujita T. (2008). Salt excess causes left ventricular diastolic dysfunction in rats with metabolic disorder. Hypertension.

[B148-pharmaceuticals-03-00940] Matsui H., Barry W.H., Livsey C., Spitzer K.W (1995). Angiotensin II stimulates sodium-hydrogen exchange in adult rabbit ventricular myocytes. Cardiovasc. Res..

[B149-pharmaceuticals-03-00940] Aviv A. (1994). Cytosolic Ca^2+^, Na^+^/H^+^ antiport, protein kinase C trio in essential hypertension. Am. J. Hypertens..

[B150-pharmaceuticals-03-00940] Schiffrin E.L., Park J.B., Intengan H.D., Touyz R.M. (2000). Correction of arterial structure and endothelial dysfunction in human essential hypertension by the angiotensin receptor antagonist losartan. Circulation.

[B151-pharmaceuticals-03-00940] de Resende M.M., Mill J.G. (2007). Effect of high salt intake on local renin-angiotensin system and ventricular dysfunction following myocardial infarction in rats. Clin. Exp. Pharmacol. Physiol..

[B152-pharmaceuticals-03-00940] Liang B., Leenen F.H. (2008). Prevention of salt-induced hypertension and fibrosis by AT1–receptor blockers in Dahl S rats. J. Cardiovasc. Pharmacol..

[B153-pharmaceuticals-03-00940] Liang B., Leenen F.H. (2007). Prevention of salt induced hypertension and fibrosis by angiotensin converting enzyme inhibitors in Dahl S rats. Br. J. Pharmacol..

[B154-pharmaceuticals-03-00940] Linz W., Schölkens B.A., Ganten D. (1989). Converting Enzyme Inhibition Specifically Prevents the Development and Induces Regression of Cardiac Hypertrophy in Rats. Clin. Exp. Hypertens..

[B155-pharmaceuticals-03-00940] Frohlich E.D. (1991). Left ventricular hypertrophy: dissociation of structural and functional effects by therapy. Adv. Exp. Med. Biol..

[B156-pharmaceuticals-03-00940] Biagini G., Zoli M., Torri C., Boschi S., Vantaggiato G., Ballestri M., Baraldi A., Agnati L. F. (1997). Protective effects of delapril, indapamide and their combination chronically administered to stroke-prone spontaneously hypertensive rats fed a high-sodium diet. Clin. Sci. (Lond.).

[B157-pharmaceuticals-03-00940] Migdalof B.H., Antonaccio M.J., McKinstry D.N., Singhvi S.M., Lan S.J., Egli P., Kripalani K.J. (1984). Captopril: pharmacology, metabolism and disposition. Drug Metab. Rev..

[B158-pharmaceuticals-03-00940] Inada Y., Tanabe M., Shibouta Y., Kawazoe K., Nishikawa K., Kikuchi S. (1986). Antihypertensive action of a non-sulfhydryl angiotensin converting enzyme inhibitor (CV-3317) in various hypertensive models. Jpn. J. Pharmacol..

[B159-pharmaceuticals-03-00940] Dunn F.G., Oigman W., Ventura H.O., Messerli F.H., Kobrin I., Frohlich E.D. (1984). Enalapril improves systemic and renal hemodynamics and allows regression of left ventricular mass in essential hypertension. Am. J. Cardiol..

[B160-pharmaceuticals-03-00940] di Nicolantonio R. (1983). Failure of captopril to lower blood pressure in spontaneously hypertensive rats offered water and saline to drink. Clin. Exp. Pharmacol. Physiol..

[B161-pharmaceuticals-03-00940] Fliser D., Nowack R., Wolf G., Ritz E. (1993). Differential effects of ACE inhibitors and vasodilators on renal function curve in patients with primary hypertension. Blood Press..

[B162-pharmaceuticals-03-00940] Bouaziz H., Joulin Y., Safar M., Benetos A. (1994). Effects of bradykinin B2 receptor antagonism on the hypotensive effects of ACE inhibition. Br. J. Pharmacol..

